# Effectiveness and cost-effectiveness of a 1-year dietary and physical activity intervention of childhood obesity—study protocol for a randomized controlled clinical trial

**DOI:** 10.1186/s13063-024-08348-7

**Published:** 2024-07-27

**Authors:** A. Martikainen, AM Eloranta, U. Schwab, T. Örmälä

**Affiliations:** 1 Clinical Nutrition, University of Helsinki and Helsinki University Hospital, Hyvinkää, Finland; 2 Institute of Public Health and Clinical Nutrition, School of Medicine, University of Eastern Finland, Kuopio, Finland; 3 Institute of Biomedicine, School of Medicine, University of Eastern Finland, Kuopio, Finland; 4 Department of Medicine, Endocrinology and Clinical Nutrition, Kuopio University Hospital, Kuopio, Finland; 5 Department of Pediatrics, Helsinki University Hospital, Hyvinkää Hospital, Hyvinkää, Finland

**Keywords:** Children, Diet, Physical activity, Obesity, Intervention, Health care, Cost

## Abstract

**Background:**

We investigate and try to find out the optimal duration and intensity for the treatment and content useful for clinical work. The aim of our study is to evaluate the effects of lifestyle intervention on the management of childhood overweight and to explore the factors that contribute to the outcome, as well as the costs for the health care system. The hypotheses of the study are that lifestyle intervention is efficient in reducing BMI-SDS and thus effective in preventing overweight from progressing to obesity, and it is also cost-effective.

**Methods and analyses:**

We aim to recruit 80 children and they randomize either to an intervention group or a control group with standard care. The intervention group receives intensive, family-based diet, and physical activity counseling, delivered by a multidisciplinary team of a pediatrician, a nurse, and a clinical nutritionist. The control group does not receive any lifestyle intervention during the study. The inclusion criteria are age of 6–12 years, weight-for-height ≥  + 40% or ≥  + 30%, and increasing curve. All participants fill out the study questionnaires and plasma samples are taken at baseline and at 12 months. Outcome variables will be compared between intervention and control groups.

**Discussion:**

If the effects of this lifestyle intervention are positive and it is also cost-effective, the implication of our study will be of great importance to the treatment of childhood obesity and to improve the health care system.

**Trial registration:**

ClinicalTrials.gov NCT06126679. Registered on 25 October 2028 in Finland.

ORCID: 0009-0009-6659-5290.

## Introduction

Obesity is the most prevalent chronic health condition in the pediatric population [1]. The number of school-aged children and adolescents with obesity has increased more than tenfold in just 40 years [2]. The costs of overweight and obesity have been increasing during the past decades and obesity has become a major global health challenge. An estimated 216 million children were overweight in 2016 [2]. In Finland, at least 25% of boys and almost 20% of girls aged 2–16 years are overweight, and 9% of boys and 4% of girls are obese [3]. If both parents are obese the child’s risk of being obese as an adult is 6- to 15-fold as compared with a child without obese parents [4, 5].

Being overweight in childhood is associated with an increased risk of obesity and many chronic diseases and other health problems such as mental and psychological issues in later life [6]. It is associated with an increased risk of hyperinsulinemia, insulin resistance, prediabetes, and subsequently type 2 diabetes in childhood. Mean arterial blood pressure increases significantly with increasing BMI in children [7]. Nonalcoholic fatty liver disease (NAFLD) is a chronic liver disease resulting from excessive fat accumulation in the liver and is strongly associated with obesity in children [8]. Obese or overweight children are also often affected by weight-related stigmatization, discrimination as well as frequent teasing by peers causing psychosocial health problems, such as low self-esteem, body dissatisfaction, eating disorders, depression, anxiety, and social isolation [9] and low health-related quality of life (HRQoL) [6, 9, 10]. The risks of health problems among overweight or obese children who become non-obese by adulthood are like those who were never obese [11].

There is also proportionality between BMI and costs for society [12]. The lifetime cost is almost three times greater per child with obesity than per child with overweight [12]. The costs of overweight and obesity in adulthood are greater the longer the person has been obese, especially if present from childhood or adolescence [12].

Clinical interventions to manage childhood obesity have the potential to reduce the prevalence of obesity in adults, improve long-term quality of life, and reduce health care costs [13, 14]. Lifestyle interventions in children, including multicomponent guiding with healthy diet, increasing physical activity, and decreasing sedentary time with behavioral change can be effective in achieving reductions in body mass index and can decrease the risk of health problems associated with obesity in adulthood [6, 15–17]. Weight loss is greater when the duration of treatment is longer than 6 months [17]. Estimated contact hours and number of sessions are the intervention components associated with effect size [18]. A meta-analysis indicates that lifestyle interventions incorporating a dietary component as a critical part of the treatment of childhood obesity led to significant weight loss [17].

Several trials have shown that pediatric obesity treatment is most effective when it starts at a younger age [6, 19, 20]. Good motivation for treatment and adherence to the protocol have contributed to a favorable outcome [20]. Lifestyle interventions are safe for children. They have few harms, and studies have found no evidence of adverse effects on growth, eating disorder pathology, or mental health [15, 21].

Further studies are needed to find out the optimal duration, intensity, and content, as well as the long-term effectiveness of dietary and physical activity interventions on overweight and obese children. There are only a few evaluations of the cost-effectiveness for the treatment of childhood overweight and obesity and more information on the cost-effectiveness of weight management interventions is needed.

We investigate the effectiveness and cost-effectiveness of a randomized 1-year dietary and physical activity intervention of childhood obesity (Hyvinkää Childhood Obesity Study, HCOS). This intervention model is in use in the treatment of children with obesity at Hyvinkää Hospital in Finland. The aim of our study is to investigate the effects of lifestyle intervention on the management of childhood overweight and the prevention of developing obesity. The second aim of our study is to study the cost-effectiveness of the intervention. The hypotheses of the study are that lifestyle intervention is efficient in reducing body mass index standard deviation score (BMI-SDS) and thus effective in preventing overweight from progressing to obesity, and it is also cost-effective. Our study will also provide more information about the lifestyle and quality of life of overweight or obese children. If we can stop excessive weight gain in childhood, we might be able to help overweight children feel better and live healthier and save society’s expenses.

## Methods and analyses

### Participants

Hyvinkää Childhood Obesity Study, HCOS is a randomized controlled clinical trial. Study participants are recruited from the primary care and community settings within the Helsinki University Hospital (HUS), Hyvinkää hospital area in Southern Finland. In schools, public health nurse recommends children and their parents to participate in this study if the child meets the inclusion criteria. In addition, we use passive recruitment methods via local newspapers and social media, flyers, and posters at schools and other community venues.

The inclusion criteria for the study are overweight (weight for height at least + 40% or ≥ 30% and it is rising) and age of 6–12 years. Exclusion criteria are diagnosed endocrine diseases and mental illnesses. Participants must be well-motivated and participate in the study with one or both parents. Eligible participants are randomized to either the intervention group or the control group with standard care using opaque sealed envelopes.

#### Calculation of the sample size

Power calculation was performed based on the assumption that there would be a difference of 0.4 in the change of body mass index standard deviation score (BMI-SDS) between the intervention and the control groups. Calculations on a difference of 0.4 SD in the change of BMI-SDS, at the level of statistical significance of 0.05 based on two-sided testing and with power of 80% and the assumption that 10% of participants will drop out in both groups the estimated sample size was 40 children per group.

Thus, we aim to recruit 80 children, targeting 40 children in the intervention group and 40 children in the control group.

## Methods

### Study visits

All participants fill out the study questionnaires and nurses measure height, weight, waist circumference, and blood pressure at baseline and at 12 months (Table 1). Participants in the intervention group have five visits to the hospital (at baseline and 3, 6, 9, and 12 months after baseline) including a total of 10 contact hours. Participants in the control group continue with the standard care in primary care. In the intervention group, all measurements of the participants are performed in the hospital. In the control group, measurements are performed in primary care. the same measurements were used in both groups. Costs in both groups will be documented. Further follow-up is possible to collect from health inspections of school health care.

**Table 1:** SPIRIT figure of content for the schedule of enrolment, interventions, and assessments

	**Study period**							
	**Enrolment**	**Allocation**	**Post-allocation**					**Close-out**
**Timepoint**^**b**^	*** − t***_**1**_	**0**	***t***_**1**_	***t***_**2**_	***t***_**3**_	***t***_**4**_	***t***_**5**_	***t***_**6**_
**Enrolment:**								
** Eligibility screen**	x							
** Informed consent**	x							
** Allocation**		x						
**Interventions:**								
*** Intervention group***			x	x	x	x	x	
*** Control group***			x				x	
**Assessments:**								
*** Anthropometric measurements***			x		X^a^		x	
*** Blood pressure***			x		X^a^		x	
*** Laboratory values***			x				x	
*** Study questionnaires***			x				x	
*** Costs of the treatment***								x

^a^Only intervention group

^b^− *t*_1_ = recruitment, *t*_1_ = baseline, *t*_2_ = 3 months, *t*_3_ = 6 months, *t*_4_ = 9 months, *t*_5_ = 12 months, *t*_6_ = after the intervention period

### Anthropometric measurements

Body height and weight are measured by specialist nurses, according to the methods generally used in Finland. Weight is measured in light underwear using a calibrated electronic scale with an accuracy of 0.1 kg as the mean of two measurements. Height is measured with an accuracy of 0.1 cm and the mean of the two measurements of barefoot height is used. ISO-BMI is calculated as weight (kg) divided by height (m) squared and BMI-SDS is computed using Finnish references [22].

Waist circumference is measured twice to an accuracy of 0.1 cm while standing and with an unstretchable measuring tape adjusted horizontally. Waist circumference is measured during expiration at mid-distance between the bottom of the rib cage and the top of the iliac crest.

### Blood pressure

Blood pressure is measured manually by a calibrated meter. The measurement protocol includes, after a 5-min rest, three measurements from the right arm in the sitting position at 2-min intervals. The mean of all three measurements is used as the systolic and diastolic blood pressures.

### Laboratory analyses

Plasma samples are taken after 12-h fasting at baseline and at 12 months (Table 1), and they are performed in the laboratory of HUS in Finland. Laboratory analyses include blood count (hemoglobin, hematocrit, total red cell count, total white cell count, and thrombocytes), plasma concentrations of total cholesterol, high-density lipoprotein (HDL) cholesterol, low-density lipoprotein (LDL) cholesterol, triglycerides, glucose, alanine transaminase (ALAT) and high-sensitivity C-reactive protein (hs-CRP), and serum concentrations of insulin, thyroxine and thyroid-stimulating hormone (TSH), and glycosylated hemoglobin (HbA1c).

### Intervention

**Fig. 1:**
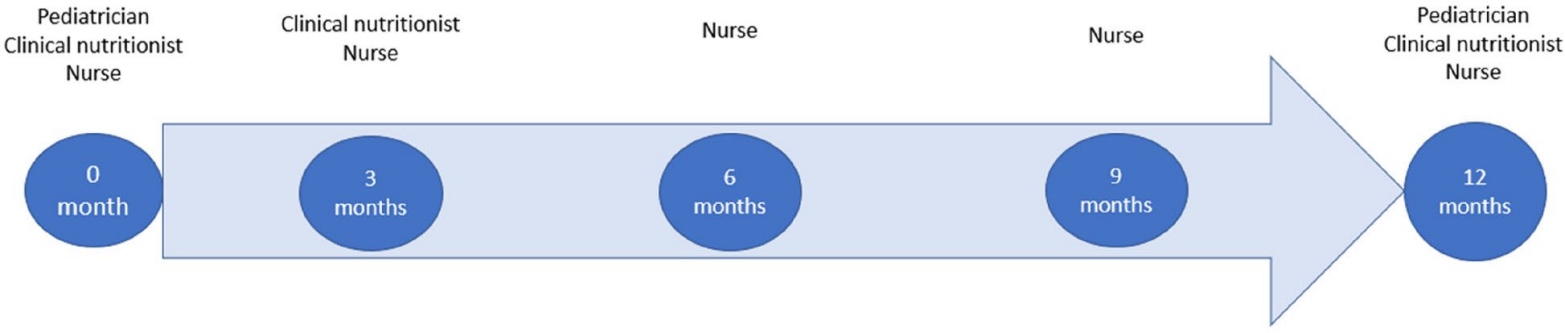
Schedule of the intervention and the participating professionals at each intervention visit

The 1-year lifestyle intervention includes intensive, family-based diet and physical activity counseling. The multidisciplinary team consists of one pediatrician, one specialist nurse, and a clinical nutritionist. Children with their parents met the pediatrician two times, the specialist nurse five times, and the clinical nutritionist three times during the 1-year intervention (Fig. 1). One of the meetings with the clinical nutritionist is only for parents and the child is with the nurse at the same time.

The treatment is based on educational and behavioral counseling and motivating the participants to change their lifestyle and to support the parents in managing their children’s behavior. The aim of counseling is to increase awareness of healthy dietary and physical activity habits; to achieve a suitable energy balance, to create a positive attitude to physical activity, to promote optimal sleep duration, and to improve the children’s body image and body control.

The pediatrician performs a clinical inspection and evaluates the laboratory analyses. The participants meet the pediatrician at the baseline and at 12 months. The special nurse measures the participant’s height, weight, waist circumference and blood pressure at the beginning of the study, at 6 months and at 12 months. The nurse advises participants to modify dietary and physical activity habits and discusses sleep duration and reducing sedentary time. Dietary and behavioral counseling is delivered by the clinical nutritionist and reinforced by the whole team. Dietary guidance is based on the Nordic and Finnish nutrition recommendations [23, 24], emphasizing appropriate energy intake, regular meal frequency, quality of dietary fat, and quality of carbohydrates, including lower sugar and higher fiber intakes. This includes increasing the consumption of vegetables, berries, fruit, sources of unsaturated fats, and wholegrain products; replacing high-fat milk and meat products with fat-free and low-fat alternatives; and reducing high-fat and high-sugar snack foods and sugar-sweetened beverages. Joint family meals are encouraged.

### Physical activity

The participants in the intervention group are advised and motivated to increase their regular daily physical activity and reduce sedentary habits. They have one group session for the training in the gymnasium with a physical education instructor. The physical education instructor gives a voluntary lecture for parents about increasing physical activity and reducing sedentary time. Unfortunately, we had to cancel the group session after the start of the COVID-19 pandemic. Nevertheless, all research visits are encouraged to increase daily activity.

The entire research team encourages and motivates the participants and their parents to carry out the instructed lifestyle changes. Families are also instructed to use Finnish lifestyle internet manuals for kids and families provided by the Finnish Heart Association. Emphasis is on educational and behavioral counseling to keep up healthy growth and development with a positive attitude.

The control group continues with the normal primary care and does not receive any special intervention during the study period. Implementing intervention will not require alteration in usual care.

## Study questionnaires

All participants fill out the study questionnaires at baseline and at 12 months. Most of the study questionnaires were developed and used in the Finnish Physical Activity and Nutrition in Children Study (PANIC) [25], and thus well noticed for this age group. The study questionnaires are sent to all participants by mail and are filled out by the parents with their child at home. In the intervention group, the study questionnaires are returned during the visits at the baseline and at 12 months. In the control group, they are returned in a return envelope by mail.

### Dietary intake

Energy and nutrient intake, food consumption, and eating frequency are assessed by a food diary. The food diary includes four predefined consecutive days (three weekdays and one weekend day), and it is administered by the parents. They are instructed to record all food and drink consumption, except drinking water, of their child by household measures and to ask their child about the food and drink consumption outside the home. Food records are reviewed by a clinical nutritionist at return. Dietary intake will be calculated by the AivoDiet software, version 2.2.0.0 (Mashie FoodTech Solutions Finland Oy, Turku, Finland).

Food consumption is also assessed by the structured Food Consumption Questionnaire, developed to complete the food diary data, and used in the Finnish PANIC study [25]. Data on the quality of usually chosen foods and the frequency of consumption of selected foods are collected.

### Eating behavior

Eating behavior is measured by the Children’s Eating Behaviour Questionnaire (CEBQ) [26] administered by the parents. An official version translated from English to Finnish is used. The questions of the questionnaire represented eight categories of eating behavior: enjoyment of food, food responsiveness, emotional overeating, desire to drink, satiety responsiveness, slowness in eating, emotional under-eating, and food fussiness. Each question offers response options from never to always on a 1 to 5 Likert scale and the mean of responses of each category are calculated and used in the analyses.

### Physical activity and sedentary time

Time spent in physical activity and electronic media time are assessed by the Physical Activity Questionnaire developed for the PANIC study [27]. Physical activity includes organized sports, supervised exercise other than sports, unsupervised physical activity, physically active school transportation, physical activity during recess, and physical education. Electronic media time contains watching television and videos, using a computer, playing video games, using mobile phones, and playing mobile games.

### Sleep, socioeconomic status, and quality of life

Participants’ sleep duration and quality are assessed by the sleep questionnaire [28]. The parents are asked how many hours their child sleeps at night, how their child usually sleeps after falling asleep, how many times their child wakes up at night, how often their child snores, and how loud it is.

Participants and their parents are also asked to fill out a background questionnaire. Children’s information includes family background, experiences of being bullied for being overweight, diagnosed disorders, medications, and vitamin and mineral supplements in use. The parent’s information includes their weight and height, marital status, and lifestyle including sleep duration and quality, dietary changes, physical activity, use of alcohol, and smoking. Socioeconomic background is assessed by the annual household income and the level of education of the parents.

HRQoL is measured by a questionnaire concerning the child’s well-being during the previous 3 months including questions on the child’s psychological, physical, and social well-being [29].

### Statistical analyses

The statistical analyses will be performed using the SPSS statistical analysis software (IBM Corporation, Armonk, NY). Baseline characteristics will be compared between the intervention and the control groups using independent samples *T* test. Changes within the intervention and the control groups during the 1-year study period will be tested using paired samples *T* test and the effectiveness of the intervention will be investigated using a general linear model for repeated measures.

### Ethics and dissemination

The study protocol of the HCOS study has been approved by the Research Ethics Committee of HUS. The participants receive both oral and written information about the study, and all participating children and their parents give their informed written consent. They have the right to discontinue the study without telling the reason. Research materials are stored in a locked and protected environment. Study results will be published in international scientific journals.

### Strengths and limitations

Most of the study questionnaires have been used before in other studies and thus well noticed for this age group and this study, this is one strength of our study. Second, one nurse measures the weight, height, waist circumference, and blood pressure of all subjects in the intervention group, which reduces measurement errors. On the other hand, there are five nurses, who measure subjects in the control group. This study includes some limitations that will limit the interpretability and generalizability of the findings. First, the intervention and control groups differ in measurements being taken by different providers in different locations, and thus, differential measurement bias could be the reason for any difference found between the groups. Second, parental involvement is required for children to enroll in and participate in the study, and thus, there lacks generalizability of any results to children who wish to enroll in a program like this but do not have the support of parents. Third, puberty staging is not evaluated. However, age and body height can be taken into account as a proxy for puberty staging in the analyses.

The most significant challenge of our study is the worldwide COVID-19 pandemic started in 2020. During the COVID-19 pandemic, the children studied at home and group activities such as physical activities were interrupted. Their eating habits and regular daily physical activity may have changed. Social relations also decreased significantly. These factors may affect the methods, study questionnaire may not give reliable information. The data on the quality of usually chosen foods and the frequency of consumption of selected foods and physical activities can be different than normal and can complicate the reliability of research reproducibility.

## Discussion

There are several previous lifestyle intervention studies, which have the potential to reduce BMI and decrease the risk of health problems associated with obesity [6, 15–17]. However, only seldom these are applicable in clinical work and there are only a few evaluations of the effectiveness with cost-effectiveness. This is why it will be of great importance to find out the optimal content, duration, and intensity of the treatment of overweight and obesity in children. Our study will provide new information on this.

Our study will provide new information about the effects and cost-effectiveness of lifestyle intervention in overweight or obese children in Finland. If the effects of the lifestyle intervention are positive and it is also cost-effective, the implication of our study will be of great importance to the treatment of childhood obesity, health care providers, and overweight or obese individuals.

The results of the present study will provide more information about the lifestyle and quality of life of overweight or obese children in Finland. If we can stop excessive weight gain in childhood, we might be able to help overweight children feel better and live healthier. Being overweight in childhood is associated with an increased risk of obesity and increased risk of many diseases and other discomforts such as mental and psychological issues [6]. Due to the high number of overweight or obese children, it will be challenging to take care of all the complications associated with obesity. However, the risk of health problems among overweight or obese children who become nonobese by adulthood is like those among persons who were never obese [11]. The lifetime cost is greater per child with obesity than per child with overweight and costs are greater the longer the person has been obese [12]. Because of these reasons, it is very important to try to stop excessive weight gain in childhood. If our lifestyle intervention is efficient in reducing BMI-SDS and cost-effective, the treatment model can be taken into wider use, and we can save society’s expenses.

## Trial status

The Hyvinkää Childhood Obesity Study (§142/2018, HUS/2666/2018) is conducted in coordination with HUS Hyvinkää hospital and community settings within the Hyvinkää area and started on 18 December 2018. The first participants started the intervention in January 2019. In early 2020, the COVID-19 pandemic started in Finland, and because of that, the recruitment of participants slowed down and was partially interrupted. The last visits of participants will be at the end of 2023 or at the latest at the beginning of 2024, and after these, the last study questionnaires will be returned. In the future, the study material will be supplemented with follow-up measurements in school health care. Because of the interruption of the COVID pandemic, the study has been delayed and this protocol article is only submitted now.

## Data Availability

The datasets analyzed during the current study and statistical code are available from the corresponding author on reasonable request, as is the full protocol.

## Declarations

This study is conducted in accordance with the ethical standards laid down in the 1964 Declaration of Helsinki and its later amendments.
